# Elizabethkingia meningoseptica Bacteremia and Meningitis: A Case Report

**DOI:** 10.7759/cureus.19275

**Published:** 2021-11-05

**Authors:** Madeeha Subhan Waleed, Vineeth Amba, Ashok Abraham Varughese, Radhika Pathalapati

**Affiliations:** 1 Internal Medicine, Ayub Medical College, Abbottabad, PAK; 2 Medicine, Rutgers Robert Wood Johnson Medical School, New Brunswick, USA; 3 Medicine, Pushpagiri Institute of Medical Sciences and Research Centre, Thiruvalla, IND; 4 Internal Medicine, Lower Bucks Hospital, Bristol, USA

**Keywords:** meningitis, chryseobacterium meningosepticum, sepsis, bacteremia, elizabethkingia meningoseptica

## Abstract

*Elizabethkingia meningoseptica* (*Chryseobacterium meningosepticum*) is a gram-negative aerobic organism with yellow pigments. Although extremely rare, human infections associated with this organism have been reported. *Elizabethkingia meningoseptica* is usually resistant to most antibiotics. Herein, we present a case of a 70-year-old woman suffering from *Elizabethkingia meningoseptica *bacteremia and meningitis. Early diagnosis and treatment are vital for an improved patient outcome. Physicians and microbiologists should be equipped with adequate knowledge regarding this infection and its treatment.

## Introduction

*Elizabethkingia meningoseptica* (*Chryseobacterium meningosepticum*) is a gram-negative aerobic organism with yellow pigments. It is a non-motile, oxidase-positive, non-spore-forming organism, and it was first described by Elizabeth O. King in 1959 [1,2]. Although extremely rare, human infections associated with this organism have been reported, causing nosocomial infections in the vulnerable populations (immunocompromised patients and neonates) and neonatal meningitis and sepsis [3]. It can also colonize the human adult population and cause pneumonia, meningitis, and endocarditis [4]. *Elizabethkingia meningoseptica* is resistant to most antibiotics [5]. Early and accurate identification of this organism helps reduce the morbidity and mortality associated with *Elizabethkingia meningoseptica* bacteremia.

## Case presentation

A 70-year-old woman presented to the emergency department (ED) with a generalized weakness for one week. Her medical history revealed low-grade marginal B-cell lymphoma with six cycles of bendamustine plus rituximab (BR) completed two years before presentation, hypertension, autoimmune hemolytic anemia, splenectomy on monthly intravenous immunoglobulin (IVIG) therapy, recent shingles infection two months before presentation with postherpetic neuralgia, and chronic diarrhea secondary to IVIG therapy. She was doing relatively well one week before the presentation. However, she started feeling weak, which gradually progressed to the point where she was unable to walk on her own; thus, she visited the ED. According to the emergency medical service, she was hypotensive (systolic blood pressure (BP) of 60 mmHg). The patient denied any other symptoms. In the ED, the patient was still hypotensive, tachycardiac, and with lactic acid of > 2 mmol/L. Her troponin levels and electrocardiogram (ECG) were normal. She received a 2 L bolus of Ringer lactate and was started in norepinephrine bitartrate, and her BP improved. She was admitted to the intensive care unit and was diagnosed with septic shock. She was started on intravenous vasopressors and antibiotics. Laboratory results are shown in Tables 1, 2.

**Table 1:** Complete blood picture

	Normal Values	On Admission	3 days post-admission	1 week post-admission	2 weeks post-admission	At discharge
Hemoglobin	Male: 13.5-17.5 g/dL Female: 12.0-16.0 g/dL	9.0 g/dL	9.3 g/dL	9.2 g/dL	9.3 g/dL	9.7 g/dL
Hematocrit	Male: 41%-53% Female: 36%-46%	27.1%	27.4%	27.6%	26.6%	26.8%
Mean Corpuscular Hemoglobulin	25.4-34.6 pg/cell	27.1 pg/cell	27.0 pg/cell	27.1 pg/cell	27.3 pg/cell	27.2 pg/cell
Mean Corpuscular Volume	80-100 µm^3^	100 µm^3^	99.6 µm^3^	100.4 µm^3^	100.4 µm^3^	99.3 µm^3^
Platelet	150-400 x 10^9^/L	100 x 10^9^/L	132 x 10^9^/L	156 x 10^9^/L	136 x 10^9^/L	103 x 10^9^/L
Neutrophil%	40-60%	19.97%	47.3%	52.4%	40.5%	40.2%
Lymphocyte%	20-40%	29.3%	24.6%	26.9%	31.5%	40.2%
Monocyte%	4-8%	23.4%	25.9%	21.1%	25.1%	18.7%
Eosinophil%	1-3%	0.00%	0.3%	0.3%	0.0%	0.0%
Basophil%	0-1%	0.00%	1.2%	1.4%	1.6%	0.9%
Immature granulocyte%	<1%	0.3%	0.6%	0.3%	0.3%	0.3%

**Table 2:** Red blood cell (RBC) morphology

Red cell morphology	Abnormality
Anisocytosis	Moderate
Macrocytosis	Moderate
Microcytosis	Slight
Hypochromia	Slight
Polychromia	Slight
Poikilocytosis	Moderate
Elliptocytosis	Slight
Ovalocytes	Slight
Schistocytes	Slight
Acanthocytes	Slight
Smudge cell	Present
Giant platelets	Present
Hyper segmentation	Present

Her blood cultures were performed, and results showed *Elizabethkingia meningoseptica* growth in the anaerobic bottle, and urine culture was positive for *Klebsiella pneumoniae*. Her cerebrospinal fluid (CSF) culture also revealed *Elizabethkingia meningoseptica* growth. She also had acute kidney injury during her admission, which resolved with shock resolution. She had a baseline creatinine of 0.8 mg/dL, which went up to 2.9 mg/dL and returned to 0.3 mg/dL at discharge, as shown in Table 3.

**Table 3:** Complete metabolic profile

	Normal Values	On admission	3 days after admission	1 week after admission	2 weeks after admission	At discharge
Sodium	135-145 milliequivalents per liter (meq/L)	133 meq/L	136 meq/L	139 meq/L	135 meq/L	137 meq/L
Potassium	3.5-5.1 meg/L	4.6 meq/L	4.6 meq/L	3.7 meq/L	3.8 meq/L	3.4 meq/L
Anion gap serum	3 to 10 meq/L	8 meq/L	8 meq/L	7 meq/L	6 meq/L	5 meq/L
Blood urea nitrogen (BUN) serum	6-20 mg/dL	15 mg/dL	12 mg/dL	8 mg/dL	9 mg/dL	8 mg/dL
Creatinine serum	0.6-1.3 mg/dL	0.8 mg/dL	2.9 mg/dL	1.1 mg/dL	0.5 mg/dL	0.3 mg/dL
Glucose	70-99 70-99 mg/dL	92 mg/dL	80 mg/dL	87 mg/dL	79 mg/dL	94 mg/dL
Protein total	6.0-8.3 grams per deciliter (g/dL)	4.4 g/dL	4.1 g/dL	4.2 g/dL	4.1 g/dL	4.1 g/dL
Albumin serum	3.4-5.4 (g/dL)	2.8 g/dL	2.7 g/dL	3.0 g/dL	2.7 g/dL	2.8 g/dL
Bilirubin total serum	Upto 1.2 mg/dL	0.7 mg/dL	1.0 mg/dL	0.9 mg/dL	0.8 mg/dL	0.6 mg/dL
Alkaline phosphatase serum	20-130 U/L	159 U/L	155 U/L	149 U/L	138 U/L	141 U/L
Aspartate Aminotransferase (AST) U/L	8-33 U/L	25 U/L	725 U/L	525 U/L	40 U/L	38 U/L
Aspartate Aminotransferase (ALT) U/L	4-36 U/L	43 U/L	188 U/L	120 U/L	42 U/L	35 U/L

The hospital stay was complicated with multiple episodes of supraventricular tachycardia of up to 160-190 beats/min, which broke spontaneously a week after she was started on amiodarone. Amiodarone was later discontinued after a week due to acute elevation in transaminases: aspartate aminotransferase (AST) of 725 U/L and alanine aminotransferase (ALT) of 188 U/L, as shown in Table 3. Echocardiography was performed at admission, showing ejection fraction (EF) of 15% with severely decreased left ventricular (LV) systolic function, grade 2 diastolic dysfunction, and mild mitral regurgitation, tricuspid regurgitation, pulmonary regurgitation, aortic stenosis peak of 5.4 m/s (mean 3.4), aortic valve area (AVA) of 1.89 cm^2^, and multiple left ventricular wall motion abnormalities (regional abnormalities in contractile function). The patient was advised cardiac catheterization for ischemia after the resolution of her infection. Her blood lactate levels gradually improved, and pressors were tapered after five days. Metabolic encephalopathy was resolved with septic shock resolution. A transesophageal echocardiogram (TEE) was performed after five days to rule out endocarditis and revealed an improved EF of 35-40%, with no evidence of valvar vegetations. A moderate-to-severe decrease in the global systolic left ventricular function was observed. A gallium scan was performed and showed a persistent increase of 2-3 intensity gallium uptakes in the left proximal femur, greater trochanter region suspected of osteomyelitis. Magnetic resonance imaging of the right hip was performed but did not show signs of osteomyelitis. The results showed left greater trochanteric bursitis, left hip osteoarthritis, and incidentally noted left superior acetabular labral tear. A lumbar puncture was performed, and cerebrospinal fluid (CSF) examination results were consistent with bacterial meningitis, as shown in Table 4.

**Table 4:** Cerebrospinal fluid findings of the patient

Pressure (cmH2O)	Appearance	Glucose (mg/dL)	Protein (mg/dL)	White Blood Cell count (cells/mm^3^)	Culture
>30	Turbid	14	90	830	Elizabethkingia meningoseptica

The antibiotic regimen was changed a couple of times due to a resistant organism. She was initially given meropenem, which was changed to meropenem and vaborbactam (Vabomere), and ciprofloxacin and piperacillin and tazobactam (Zosyn) were added a day later, and then Bactrim was also added. Sensitivity revealed multidrug-resistant organisms. She was started on levofloxacin, minocycline, and rifampin two days later. A peripherally inserted central catheter was placed to administer antibiotics. Blood cultures were repeated daily and showed negative results two days after starting the above regimen and remained negative for three days. The patient was discharged on levofloxacin, minocycline, and rifampin for a total of four weeks from the first negative culture. Acyclovir was continued for antiviral prophylaxis. At discharge, the patient was afebrile, hemodynamically stable, and advised for outpatient primary care physician, infectious disease, cardiology, gastroenterology, and hematology/oncology follow-up.

## Discussion

*Elizabethkingia meningoseptica* is an uncommon organism primarily affecting neonates and immunocompromised patients. Clinical data regarding *Elizabethkingia meningoseptica* infections remain limited. This organism is intrinsically resistant to several antibiotics [6]. Our patient's infection also did not respond to multiple antibiotics. Literature is available regarding *Elizabethkingia meningoseptica* causing pneumonia, meningitis, soft tissue infection, and osteomyelitis. Our patient had meningitis and sepsis associated with the organism. *Elizabethkingia meningoseptica* primarily affects immunocompromised hosts and is associated with increased mortality [3]. Sepsis caused by *Elizabethkingia meningoseptica *has been reported in immunocompetent individuals [5,7]. Potential risk factors for acquiring the infection include diabetes, steroids, organ transplant, and malignancy [3]. Our patient was asplenic and also had marginal B-cell lymphoma.* Elizabethkingia meningoseptica* is particularly resistant to many antimicrobials, with rifampin potentially an effective medication [8,9]. Recent studies have also demonstrated the benefits of fluoroquinolone against the bacterium [10]. Our patient was also treated with levofloxacin, minocycline, and rifampin. Her blood cultures turned negative two days after starting the treatment.

## Conclusions

*Elizabethkingia *rarely causes human infections but has a marked antimicrobial resistance profile. Inappropriate antimicrobial therapy increases resistance and mortality in patients; therefore, effective treatment should be administered using reliable antimicrobial susceptibility testing. Early diagnosis and treatment are vital for an improved patient outcome. Currently, no drug of choice has been available for patients with *Elizabethkingia *infection, and clinical trials regarding the treatment are still lacking. Physicians should be vigilant, and once the organism is identified, appropriate antibiotics should be administered in time to reduce morbidity. Further studies regarding the epidemiology of the organism, risk factors for the infection, and appropriate treatment should be conducted. No vaccine data are available for this organism. Physicians and microbiologists should be equipped with adequate knowledge regarding this infection and how to treat it.
